# Chromosome-level genome assembly of the Tyrrhenian tree frog (*Hyla sarda*)

**DOI:** 10.1038/s41597-025-05760-9

**Published:** 2025-09-02

**Authors:** Josephine R. Paris, Roberta Bisconti, Andrea Chiocchio, Linelle Abueg, Dominic E. Absolon, Tatiana Tilley, Nivesh Jain, Jennifer Balacco, Brian O’Toole, Erich D. Jarvis, Giulio Formenti, Daniele Salvi, Daniele Canestrelli

**Affiliations:** 1 Department of Health, Life and Environmental Sciences, University of L’Aquila, Coppito, Italy; 2 Department of Life and Environmental Sciences, Marche Polytechnic University, Ancona, Italy; 3 Department of Ecological and Biological Science, Tuscia University, Viterbo, Italy; 4 Vertebrate Genome Laboratory, The Rockefeller University, New York, NY USA; 5 Wellcome Sanger Institute, Wellcome Genome Campus, Hinxton, UK

**Keywords:** Evolution, Ecology

## Abstract

The Tyrrhenian tree frog (*Hyla sarda*) is a small cryptically coloured amphibian found in Corsica, Sardinia, and the Tuscan Archipelago. Investigation into the species’ evolutionary history has revealed phenotypic changes triggered by glaciation-induced range expansion, but understanding the genetic basis of this trait variation has been hampered by the lack of a reference genome. To address this, we assembled a chromosome-level genome of *Hyla sarda* using PacBio HiFi long reads, Bionano optical maps, and Hi-C data. The assembly comprises 13 assembled chromosomes, spanning a total length of 4.15 Gb with a scaffold N50 of 385 Mb, a BUSCO completeness of 94.60%, and a *k*-mer completeness of 98.30%. Approximately 75% of the genome consists of repetitive elements. We annotated 22,847 protein-coding genes with a BUSCO completeness of 94.60% and an OMArk completeness of 93.74%. This high-quality assembly provides a valuable resource for studying phenotypic evolution and its genomic basis during range expansion, and will assist future investigations into the population and conservation genomics of *Hyla sarda*.

## Background & Summary

The Tyrrhenian tree frog, *Hyla sarda* (De Betta, 1853), is a relatively small (38–40 mm) anuran species belonging to the tree frog family Hylidae. *H. sarda* is endemic to the islands of Sardinia, Corsica, and the Tuscan archipelago (in the western Mediterranean Sea^1^), and is the sister species of the European tree frog (*Hyla arborea*). *H. sarda* is found in temperate forest and shrubland, and breeds annually from spring to summer in a variety of lentic freshwater environments such as ponds and pools. It remains near the breeding sites during most of the year^2^. Although considered a common and widespread species and listed as Least Concern by the IUCN, the species may be threatened by the reduction of natural habitat^3^. The species’ present-day distribution and distinctive biogeographic history make it an ideal model for investigating the phenotypic legacies of past biogeographical events and the underlying genomic mechanisms.

During the last glacial period, the Tyrrhenian tree frog underwent a spatial diffusion event from northern Sardinia to Corsica, promoted by the formation of a temporary and wide land-bridge between these islands, and from Corsica subsequently reached the Tuscan Archipelago via jump dispersal^4–6^. This two-step dispersal range expansion offers the rare opportunity to explore the interplay between, and long-term impact of, neutral and non-neutral processes during historical range expansion events. Recent studies have dissected the phenotypic patterns of variation of *H. sarda*, along the historical range expansion route, revealing considerable phenotypic evolution along both the south-to-north axis of the expansion, and along the route of the jump dispersal event. Specifically, *H. sarda* from the newly colonized area in Corsica exhibited larger body sizes than those in the source area in Sardinia, longer limbs, greater efficiency in jumping and adhesion, and shyer and more prudent exploration behaviour^7^, higher ability to change colour^8^, and different rates of physiological ageing^9^ and telomere dynamics^10^. On the other hand, *H. sarda* sampled from Elba Island (*i.e*., the island colonised by jump dispersal) were bolder, and less performant in jumping and adhesion, as compared to individuals from Corsica^11^. Together, these findings suggest that during post-glacial range expansions, newly established populations could have been founded by non-random samples of the phenotypic makeup of the source populations, and that different forms of dispersal might imprint distinct directions to phenotypic evolution. However, the genomic architecture of the observed phenotypic diversity remains unexplored, hindering a mechanistic understanding of the phenotypic evolution during range expansion.

Due to their distinct biological and evolutionary characteristics, assembling amphibian genomes present unique challenges and opportunities compared to the assembly of other vertebrates. Amphibians often have large and complex genomes, due primarily to a high proportion of repetitive elements^12^. Amongst the published anuran chromosome-level genome assemblies, genome spans vary from 988 Mb in the plains spadefoot toad (*Spea bombifrons*) to 10.2 Gb in the mountain yellow-legged frog (*Rana muscosa*) (Table S1)^13^. Despite their highly repetitive nature, chromosome number variation among anurans is limited, with the majority of cytological and genome assemblies demonstrating a karyotype of *2n* = 10–12 chromosomes^13,14^. Chromosomes are highly syntenic across species, as contemporary chromosome structure is derived from 13 ancestral chromosomes^15^. Due to drastic heterochiasmy, anuran sex-chromosome evolution is highly dynamic^16^, and although all *Hyla* tree frogs typically have a homomorphic X/Y sex chromosome system, it has recently been reported that *H. sarda* has Z/W sex determination^17^. The generation of a high-quality genome assembly for *Hyla sarda* will assist future investigations into sex chromosome evolution in *Hyla* tree frogs. To date, 35 anuran chromosome-level genomes have been assembled^13^ (Table S1). No genome assemblies have yet been produced for the *Hyla* genus, although assemblies for the common tree frog (*Hyla arborea*) and Savingny’s tree frog (*Hyla savignyi*) are in progress.

**Fig. 1:**
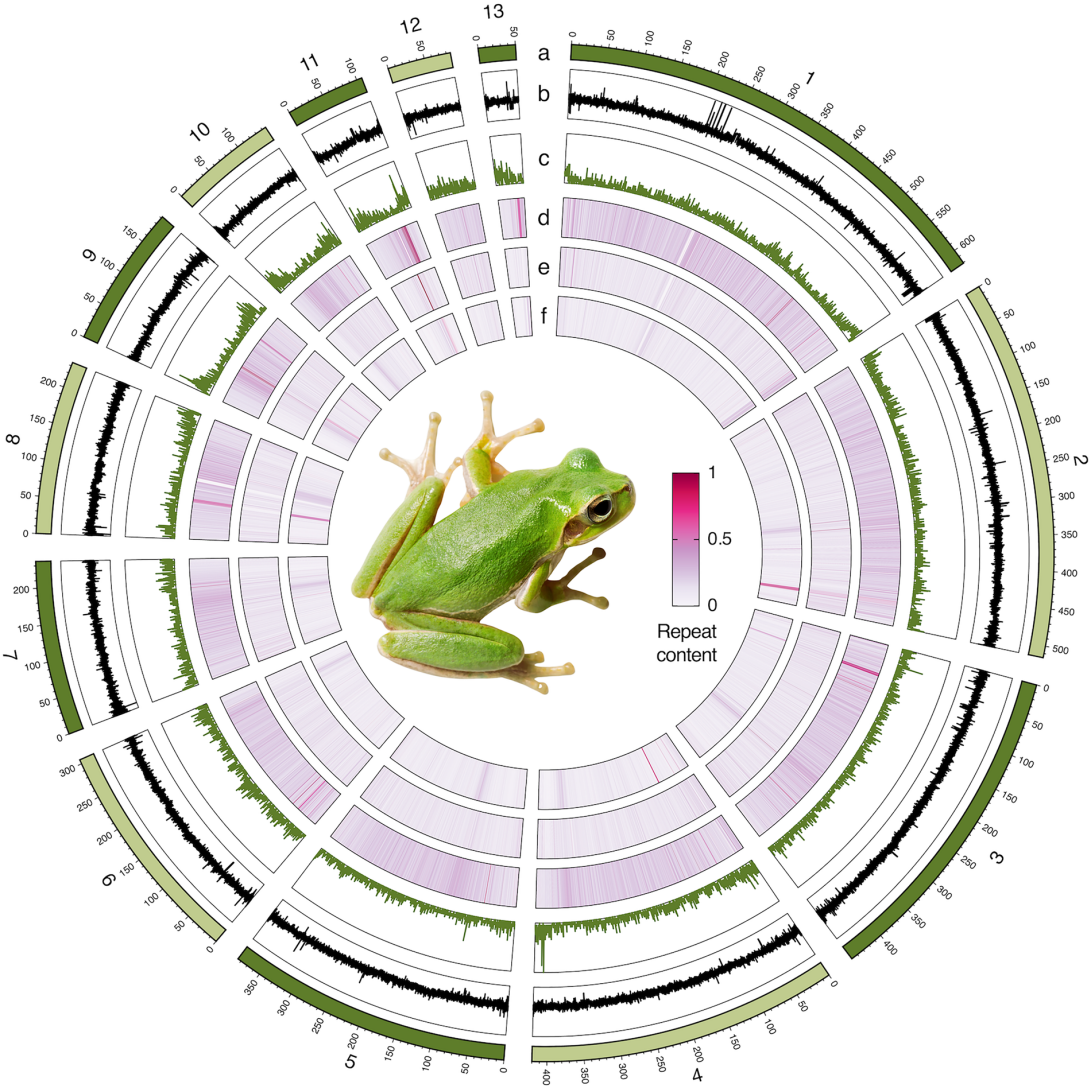
Genomic features of the Tyrrhenian tree frog (*Hyla sarda*). Circos plot of genome characteristics, showing (from the outside to the inside): (**a**) Chromosome ideograms; (**b**) GC content in 50 kb windows; (**c**) Protein-coding gene content in 200 kb windows; (**d**) DNA transposon content in 50 kb windows; (**e**) LTR content in 50 kb windows; (**f**) LINE content in 50 kb windows. See Tables 1–3 for more detailed statistics.

This study presents a chromosome-level genome assembly (Fig. 1) of the Tyrrhenian tree frog, *Hyla sarda*, assembled as part of the Vertebrate Genomes Project (VGP)^18^ using PacBio HiFi sequencing, Bionano optical maps and Arima Hi-C technology. The assembled genome spans 4.1 Gb, with a scaffold N50 of 385 Mb, and comprises 13 chromosomes and 3,412 unplaced scaffolds (Table 1). The assembly is high-quality with a BUSCO completeness of 94.60% and a *k*-mer completeness of 98.29%. A total of 74.94% (3.1 Gb) of the genome comprises repetitive sequences (Table 2), and 22,847 protein-coding genes were predicted (Table 3), with a BUSCO completeness of 94.60% and an OMArk completeness of 93.74%. This high-quality assembly and accompanying annotation will serve as a valuable resource for investigating the species’ evolutionary history, uncovering the genetic signatures of phenotypic change during range expansion, and future population and conservation genomics studies.

**Table 1:** Genome assembly statistics.

Quality metric	aHylSar1.pri.cur
Estimated genome size (Gb)	3.75
Size of final assembly (Gb)	4.15
Number of chromosomes	13
Number of scaffolds	3,474
Scaffold N50 (Mb)	384.97
Scaffold L50	5
Average scaffold length (Mb)	1.20
Largest scaffold (Mb)	620.77
Number of gaps	2,208
Total gap length	441,427
GC content	43.90
Base pair QV (k = 21)	59.88
K-mer completeness (k = 21)	98.29
BUSCO	C:94.60%[S:92.80%,D:1.70%],F:0.70%,M:4.8%

**Table 2:** Repeat annotation statistics.

Repeat class	Number of elements	Length occupied (bp)	Percentage of genome (%)
SINEs	47,523	23,365,018	0.56
LINEs	528,188	367,712,577	8.88
LTR elements	510,849	562,363,598	13.58
DNA transposons	1,680,925	940,656,455	22.71
Rolling Circle	1,020	574,408	0.01
Penelope	250,362	139,669,951	3.37
Unclassified	2,225,705	867,356,300	20.94
Other (Simple repeat, Microsatellite, RNA)	351,780	198,324,622	4.79

**Table 3:** Gene annotation statistics.

Type	aHylSar1.pri.cur
Number of protein-coding genes	22,847
Number of non-coding genes	61,641
Number of fully-supported protein-coding mRNAs	56,007
Number of fully-supported non-coding mRNAs	9,435
Number of exons in protein-coding transcripts	261,311
Number of exons in non-coding transcripts	34,666
Mean gene length (bp)	23,502
Median gene length (bp)	191
Mean mRNA transcript length (bp)	3,317
Median mRNA transcript length (bp)	2,605
Mean number of transcripts per gene	1.58
Mean number of exons per transcript	7.50
Mean exon length (bp)	295
BUSCO	C:94.60%[S:92.80%,D:1.70%],F:0.70%,M:4.80%
OMArk	C:93.74%[S:90.96%,D:2.78%],M:6.26%

## Methods

### Sample collection, extraction, and sequencing

The genome sample was obtained from an adult female collected in mainland Corsica in 2018. Biological tissue (hind leg muscle and whole brain) was flash-frozen and stored at −80 °C. Sampling procedures were performed under the approval of the Prefét de la Corse-du-Sud (#2A20180206002 and #2B20180206001). RNA-seq data derived from the brain tissue of nine individuals^19^ was used for genome annotation. Whole brain tissue was stored in RNAprotect Tissue Reagent (Qiagen) and stored at −20 °C.

For the long-read PacBio HiFi sequencing, high molecular weight (HMW) DNA was isolated from skeletal muscle using the MagAttract HMW DNA Kit (Qiagen 67563). A total of 124 mg of frozen tissue was disrupted with a Qiagen TissueRuptor II (Cat. No. 9002755). After the tissue homogenization, lysis and subsequent DNA isolation was performed following the protocol described in the MagAttract HMW DNA Handbook (Manual Purification of High-Molecular-Weight Genomic DNA from Fresh or Frozen Tissue). The purified DNA was eluted in 100 µL of Qiagen Buffer AE. The DNA was quantified with triplicate measures using a Qubit 3 fluorometer (Invitrogen Qubit dsDNA Broad Range Assay cat no. Q32850). Prior to PacBio library preparation, the DNA was sheared using the Megaruptor 3 (Diagenode, Denville, NJ, USA). HiFi libraries were prepared using the SMRTbell Express Template Prep Kit 2.0 following the manufacturer’s protocol (Pacific Biosciences, Menlo Park, CA, USA). Size-selection was performed with a Pippin HT (Sage Science, Beverly, MA, USA). The libraries were sequenced on a PacBio Sequel IIe, with Sequencing Plate 2.0 and 8 M SMRT cells, generating a total of 129 Gbp of data (~31X coverage).

For the Bionano optical map libraries, HMW DNA was extracted from skeletal muscle using the Circulomics Nanobind Tissue Big DNA Kit (PacBio, CA, USA). The DNA was quantified using the Qubit 3 fluorometer (Invitrogen Qubit dsDNA Broad Range Assay cat no. Q32850) and fragment size was assessed with a pulsed field gel electrophoresis (Pippin Pulse, SAGE Science, Beverly, MA, USA). 750 ng DNA was labelled using direct labelling enzyme (DLE1) and the Bionano Prep Direct Label and Stain (DLS) protocol (document number 30206) and then imaged on a Bionano Saphyr instrument, generating 790 MiB of data (~130X coverage).

For the Hi-C libraries, 28 mg of skeletal muscle was used for the Arima Genomics crosslinking reaction following the manufacturer’s low input sample amount guidance (Arima High Coverage HiC Kit Document Part Number: A160162). Libraries were prepared using the Arima-HiC 2.0 kit (Arima Genomics, CA, USA). The library was sequenced with the Illumina NovaSeq. 6000 platform with 150 bp paired-end reads, generating a total of 233 Gbp of data (~56X coverage).

For the genome annotation, RNA was extracted using the RNeasy Plus Kit (Qiagen), following manufacturer instructions. RNA quality and concentration were evaluated using an Agilent Cary60 UV-vis and a Bioanalyzer Agilent 2100 (Agilent Technologies, Santa Clara, CA, USA). Library preparation and sequencing were performed at NovoGene (UK). Libraries were 150 bp paired-end sequenced on an Illumina NovaSeq. 6000, generating a total of 986 Mbp of data. Further information on the RNA-seq samples can be found in Libro *et al*. 2022^19^.

### Genome assembly

The genome was assembled using the VGP v2.1 Galaxy pipeline^20^. Prior to assembly, we estimated the genome parameters with *k*-mer profiling, counting *k*-mers using Meryl^21^ and analysing the profile with GenomeScope v2^22^. Using a *k*-mer size of 21 (ploidy = 2), the estimated haploid span was 3.75 Gb, with a heterozygosity of 1.08%. Notably, *k*-mer profiling revealed a highly repetitive genome, with a repeat length of 2.3 Gb. Direct C-value estimates for *Hyla arborea* indicate a C value of 4.76 Gb (2.4–7.0).

**Fig. 2:**
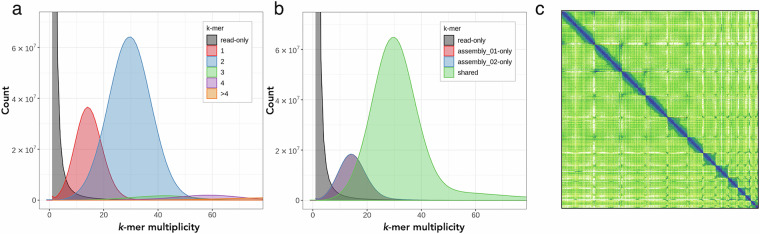
Genome assembly characteristics of the Tyrrhenian tree frog (*Hyla sarda*). (**a**) Copy number (CN) distribution plot of *k*-mer multiplicity, coloured by the number of times each *k*-mer is found in the assembly, where grey represents read-only *k*-mers, red represents one-copy *k*-mers, blue represents two-copy *k*-mers, green represents three-copy *k*-mers, purple represents four-copy *k*-mers, and orange represents five-and-more-copy *k*-mers. (**b**) Assembly (ASM) distribution plot of *k*-mer multiplicity, coloured according to which assembly contains the *k*-mers, where grey represents *k*-mers found only in the reads, red represents the primary haplotype assembly *k*-mers, blue represents the alternate haplotype assembly *k*-mers, and green represents the shared *k*-mers found in both assemblies. (**c**) PretextMap image of the 13 scaffolded pseudo-chromosomes of assembly 1 after curation (aHylSar1.pri.cur).

HiFi sequences and Hi-C data were used as input to assemble phased contigs using HiFiasm v0.16.1 in Hi-C mode^23^. The resulting haplotypes were scaffolded using the Bionano and Hi-C contact data. Bionano scaffolding was achieved using Bionano Solve v3.7.0^24^ with default parameters and without contig breaking. Hi-C scaffolding was performed on the Bionano scaffolds. Hi-C reads were aligned and prepared for scaffolding using the Arima mapping pipeline, which employs bwa mem^25^ and samtools^26^ for mapping and filtering. Scaffolding was performed using YaHS v1.2^27^. PretextMap (github.com/wtsi-hpag/PretextMap) was used to visualise Hi-C contacts before and after scaffolding. Scaffolding with Bionano and Hi-C data improved the assembly N50 from 3.88 Mbp to 417.68 Mbp. The primary haplotype was manually curated using PretextView (github.com/sanger-tol/PretextView) to correct potential assembly structural errors, to manually join and align unplaced scaffolds, and to name chromosomes^28^. We obtained a final chromosome-level genome assembly of 4.15 Gb (Table 1), which was curated into 13 chromosomes (Fig. 2C) ranging from 620.7 Mb to 50.27 Mb^29^. The final assembly span (4.15 Gb) exceeds the *k*-mer estimate of 3.75 Gb, reflecting the genome’s high repeat content. Assessment of the *k*-mer copy-number distribution confirmed that the *H. sarda* is diploid and revealed a diploid sequencing coverage of 30X and a haploid coverage of 15X (Fig. 2A). Assessment of the *k*-mer distribution between the primary haplotype and alternate haplotype assemblies revealed that diploid regions are shared by both assemblies and evidenced a high overlap between the haploid coverage *k*-mers (Fig. 2B). Genomic features were visualized using Circos^30^.

### Transposable element annotation

For transposable element (TE) annotation, we used the EarlGrey TE annotation pipeline, which has been shown to increase TE consensus sequence length and resolve spurious overlapping and fragmented annotations^31^. EarlGrey v5.0.0 was run using RepeatModeler v2.0.6^32^ and RepeatMasker v4.1.7^33^ with NCBI/RMBLAST 2.10.0 + against the Dfam v3.8^34^ Sarcopterygii partition and the Repbase RepeatMasker edition (version 20181026)^35^ libraries. Spurious TE annotations < 100 bp were removed. In total, 3.1 Gb of repetitive sequence was detected, constituting 74.94% of the *H. sarda* genome assembly. DNA transposons were the predominant family, spanning 941 Mb (22.71%), followed by LTR retrotransposons (562 Mb; 13.58%), LINEs (368 Mb; 8.88%), and SINEs (23 Mb; 0.56%) (Table 2). In addition, 867 Mb (20.94%) of repeats remained unclassified, indicating the presence of potentially novel repeat families that warrant further investigation in the context of the species’ phenotypic evolution.

### Gene prediction and functional annotation

Gene prediction and functional annotation was performed by the National Center for Biotechnology Information (NCBI) using the NCBI Eukaryotic Genome Annotation Pipeline^36^ on the aHylSar1.pri.cur assembly^29^. To assess annotation quality, BUSCO v4.1.4^37,38^ analysis was performed, using the tetrapoda_odb10 (n = 5310) OrthoDb v10^39^ lineage dataset, and OMArk v0.3.0^40^ using OMAmer v2.0.3 was run using the Tetrapoda clade (11,140 HOGs), using the longest isoform of each protein. In total, 102,483 genes and pseudogenes were predicted, including 22,847 protein-coding genes (56,007 fully-supported mRNAs), and 65,576 non-coding RNAs (see Table 3 for complete annotation statistics).

### Mitogenome assembly and annotation

The mitogenome was assembled using MitoHifi v2^41^, using MitoFinder v1.4.2^42^ for annotation. The mitogenome of *Hyla annectans* (KM271781.1)^43^ was used as the starting sequence. The resulting circularised mitogenome was 18,195 bp in length and contained the standard 37 vertebrate mitochondrial genes (13 protein-coding, 22 tRNAs, and 2 rRNAs).

## Data Records

Raw sequencing and mapping data are available from the VGP GenomeArk repository (https://genomeark.github.io/genomeark-all/Hyla_sarda.html) and on the NCBI/ENA under BioProject: PRJNA1294985 (https://www.ncbi.nlm.nih.gov/bioproject/PRJNA1294985).

The primary genome assembly (aHylSar1.hap1) is available at NCBI GenBank under the accession GCF_029499605.1^29^. It is also available in Ensembl Rapid Release (https://rapid.ensembl.org/Hyla_sarda_GCA_029499605.1/Info/Index) and the UCSC Genome Browser (https://genome.ucsc.edu/h/GCF_029499605.1).

The alternate haplotype (aHylSar1.hap2) is available at NCBI GenBank under the accession GCA_029493135.1^44^. It is also available in the UCSC Genome Browser (https://genome.ucsc.edu/h/GCA_029493135.1).

The mitochondrial genome sequence is available in NCBI GenBank, accession CM056048.1^45^.

## Technical Validation

Quality and completeness of the assembly was performed at every step of the assembly process using with Merqury v1.3^21^, gfastats^46^, BUSCO v5.3.0^37,38^ with the tetrapoda_odb10 (n = 5,310) OrthoDb v10^39^ dataset. The BUSCO completeness is 94.60% complete (92.80% as single-copy, 1.70% as duplicated), 0.40% fragmented, 4.80% missing. The Merqury *k*-mer assessment revealed a QV score of 59.88 and a completeness of 98.29%. We found that the majority (91%) of the assembled genome is contained within the 13 largest scaffolded chromosomes confirmed by Hi-C analysis. The assembly contains 2,208 gaps. Telomeric repeat sequences, identified using tidk v0.2.31^47^, were found to be enriched on at least one end of 8 of the 13 chromosomes.

To assess annotation completeness, we also used BUSCO analysis (as above) in protein mode. We also performed assessment using OMArk v0.3.0^40^ (Tetrapoda, n = 11,140 HOGs), identifying 93.74% complete HOGs (of which 90.96% are single-copy and 2.78% are duplicated (0.47% expected, 2.32% unexpected), and which 6.26% are missing. With OMArk, the proteome showed a 92.59% consistent lineage placement (9.84% partial hits, 2.07% fragmented). No contamination was identified.

## Supplementary information

Supplementary Table 1

## Data Availability

All software and pipelines were executed according to the methods section. No custom code was generated for this study.

## References

[1] Bernini, F., Doria, G., Razzetti, E. & Sindaco, R. *Atlas of Italian Amphibians and Reptiles*. (Societas Herpetologica Italica, Polistampa, 2006).

[2] Lanza, B., Andreone, F., Bologna, M. A., Corti, C. & Razzetti, E. *Amphibia. Fauna d’Italia* (Calderini, 2007).

[3] Romano, A. *et al*. *Hyla sarda*. The IUCN Red List of Threatened Species 2024, e.T55645A223764163 10.2305/IUCN.UK.2024-2.RLTS.T55645A223764163.en (2023).

[4] Bisconti, R., Canestrelli, D. & Nascetti, G. Genetic diversity and evolutionary history of the Tyrrhenian treefrog *Hyla sarda* (Anura: Hylidae): adding pieces to the puzzle of Corsica-Sardinia biota. *Biological Journal of The Linnean Society* **103**, 159–167, 10.1111/j.1095-8312.2011.01643.x (2011).

[5] Bisconti, R., Canestrelli, D., Colangelo, P. & Nascetti, G. Multiple lines of evidence for demographic and range expansion of a temperate species (*Hyla sarda*) during the last glaciation. *Mol. Ecol.* **20**, 5313–5327, 10.1111/j.1365-294X.2011.05363.x (2011). 22097966 10.1111/j.1365-294X.2011.05363.x

[6] Spadavecchia, G., Chiocchio, A., Bisconti, R. & Canestrelli, D. *Paso doble*: A two-step Late Pleistocene range expansion in the Tyrrhenian tree frog *Hyla sarda*. *Gene* **780**, 145489, 10.1016/j.gene.2021.145489 (2021). 33588038 10.1016/j.gene.2021.145489

[7] Bisconti, R., Chiocchio, A., Costantini, D., Carere, C. & Canestrelli, D. Drivers of phenotypic variation along a Late Pleistocene range expansion route. J. Biogeogr.e70044, 10.1111/jbi.70044 (2025).

[8] Spadavecchia, G. *et al*. Spatial differentiation of background matching strategies along a Late Pleistocene range expansion route. *Evol. Ecol.* **37**, 291–303, 10.1007/s10682-022-10216-2 (2023).

[9] Liparoto, A., Canestrelli, D., Bisconti, R., Carere, C. & Costantini, D. Biogeographic history moulds population differentiation in ageing of oxidative status in an amphibian. *J. Exp. Biol.* **223**, jeb235002, 10.1242/jeb.235002 (2020). 32978316 10.1242/jeb.235002

[10] Canestrelli, D. *et al*. Biogeography of telomere dynamics in a vertebrate. *Ecography (Cop.)* **44**, 453–455, 10.1111/ecog.05286 (2021).

[11] Bisconti, R. *et al*. Evolution of personality and locomotory performance traits during a Late Pleistocene island colonization in a tree frog. *Curr. Zool.* **69**, 631–641, 10.1093/cz/zoac062 (2023). 37637312 10.1093/cz/zoac062 PMC10449429

[12] Kosch, T. A. *et al*. Comparative analysis of amphibian genomes: An emerging resource for basic and applied research. *Mol. Ecol. Resour.* **25**, e14025, 10.1111/1755-0998.14025 (2025). 39364691 10.1111/1755-0998.14025 PMC11646304

[13] Challis, R., Kumar, S., Sotero-Caio, C., Brown, M. & Blaxter, M. Genomes on a Tree (GoaT): A versatile, scalable search engine for genomic and sequencing project metadata across the eukaryotic tree of life. *Wellcome Open Res* **8**, 24, 10.12688/wellcomeopenres.18658.1 (2023). 36864925 10.12688/wellcomeopenres.18658.1 PMC9971660

[14] Morescalchi, A. Evolution and karyology of the amphibians. *Boll. Zool.* **47**, 113–126, 10.1080/11250008009438709 (1980).

[15] Bredeson, J. V. *et al*. Conserved chromatin and repetitive patterns reveal slow genome evolution in frogs. *Nat. Commun.* **15**, 579, 10.1038/s41467-023-43012-9 (2024). 38233380 10.1038/s41467-023-43012-9 PMC10794172

[16] Jeffries, D. L. *et al*. A rapid rate of sex-chromosome turnover and non-random transitions in true frogs. *Nat. Commun.* **9**, 4088, 10.1038/s41467-018-06517-2 (2018). 30291233 10.1038/s41467-018-06517-2 PMC6173717

[17] Dufresnes, C., Brelsford, A., Baier, F. & Perrin, N. When sex chromosomes recombine only in the heterogametic sex: Heterochiasmy and heterogamety in *Hyla* tree frogs. *Mol. Biol. Evol.* **38**, 192–200, 10.1093/molbev/msaa201 (2021). 32761205 10.1093/molbev/msaa201 PMC7782862

[18] Rhie, A. *et al*. Towards complete and error-free genome assemblies of all vertebrate species. *Nature* **592**, 737–746, 10.1038/s41586-021-03451-0 (2021). 33911273 10.1038/s41586-021-03451-0 PMC8081667

[19] Libro, P. *et al*. First brain *de novo* transcriptome of the Tyrrhenian tree frog, *Hyla sarda*, for the study of dispersal behavior. *Front. Ecol. Evol.* **10**, 947186, 10.3389/fevo.2022.947186 (2022).

[20] Larivière, D. *et al*. Scalable, accessible and reproducible reference genome assembly and evaluation in Galaxy. *Nat. Biotechnol.* **42**, 367–370, 10.1038/s41587-023-02100-3 (2024). 38278971 10.1038/s41587-023-02100-3 PMC11462542

[21] Rhie, A., Walenz, B. P., Koren, S. & Phillippy, A. M. Merqury: reference-free quality, completeness, and phasing assessment for genome assemblies. *Genome Biol.* **21**, 245, 10.1186/s13059-020-02134-9 (2020). 32928274 10.1186/s13059-020-02134-9 PMC7488777

[22] Ranallo-Benavidez, T. R., Jaron, K. S. & Schatz, M. C. GenomeScope 2.0 and Smudgeplot for reference-free profiling of polyploid genomes. *Nat. Commun.* **11**, 1432, 10.1038/s41467-020-14998-3 (2020). 32188846 10.1038/s41467-020-14998-3 PMC7080791

[23] Cheng, H., Concepcion, G. T., Feng, X., Zhang, H. & Li, H. Haplotype-resolved *de novo* assembly using phased assembly graphs with hifiasm. *Nat. Methods* **18**, 170–175, 10.1038/s41592-020-01056-5 (2021). 33526886 10.1038/s41592-020-01056-5 PMC7961889

[24] Bocklandt, S., Hastie, A. & Cao, H. Bionano genome mapping: High-throughput, ultra-long molecule genome analysis system for precision genome assembly and haploid-resolved structural variation discovery. in *Single molecule and single cell sequencing*. Advances in Experimental Medicine and Biology, vol 1129 (ed. Suzuki, Y.) 97-118 10.1007/978-981-13-6037-4_7 (Springer, Singapore, 2019). 10.1007/978-981-13-6037-4_7 30968363

[25] Li, H. Aligning sequence reads, clone sequences and assembly contigs with BWA-MEM. arXiv:1303.3997 10.48550/arXiv.1303.3997 (2013).

[26] Li, H. *et al*. The Sequence Alignment/Map format and SAMtools. *Bioinformatics* **25**, 2078–2079, 10.1093/bioinformatics/btp352 (2009). 19505943 10.1093/bioinformatics/btp352 PMC2723002

[27] Zhou, C., McCarthy, S. A. & Durbin, R. YaHS: yet another Hi-C scaffolding tool. *Bioinformatics* **39**, btac808, 10.1093/bioinformatics/btac808 (2023). 36525368 10.1093/bioinformatics/btac808 PMC9848053

[28] Howe, K. *et al*. Significantly improving the quality of genome assemblies through curation. *Gigascience* **10**, giaa153, 10.1093/gigascience/giaa153 (2021). 33420778 10.1093/gigascience/giaa153 PMC7794651

[29] Vertebrate Genomes Project & NCBI RefSeq *Hyla sarda* genome assembly aHylSar1.hap1. *NCBI GenBank* http://identifiers.org/assembly:GCF_029499605.1 (2023)

[30] Krzywinski, M. *et al*. Circos: an information aesthetic for comparative genomics. *Genome research* **19**, 1639–1645, http://www.genome.org/cgi/doi/10.1101/gr.092759.109 (2009). 19541911 10.1101/gr.092759.109 PMC2752132

[31] Baril, T., Galbraith, J. & Hayward, A. Earl Grey: A fully automated user-friendly transposable element annotation and analysis pipeline. *Mol. Biol. Evol.* **41**, msae068, 10.1093/molbev/msae068 (2024). 38577785 10.1093/molbev/msae068 PMC11003543

[32] Flynn, J. M. *et al*. RepeatModeler2 for automated genomic discovery of transposable element families. *Proc. Natl. Acad. Sci. USA.* **117**, 9451–9457, 10.1073/pnas.1921046117 (2020). 32300014 10.1073/pnas.1921046117 PMC7196820

[33] Smit, A. F. A., Hubley, R. & Green, P. *RepeatMasker Open-4.0. 2013-2015*. (2015).

[34] Storer, J., Hubley, R., Rosen, J., Wheeler, T. J. & Smit, A. F. The Dfam community resource of transposable element families, sequence models, and genome annotations. *Mob. DNA* **12**, 1–14, 10.1186/s13100-020-00230-y (2021). 33436076 10.1186/s13100-020-00230-y PMC7805219

[35] Bao, W., Kojima, K. K. & Kohany, O. Repbase Update, a database of repetitive elements in eukaryotic genomes. *Mob. DNA* **6**, 1–6, 10.1186/s13100-015-0041-9 (2015). 10.1186/s13100-015-0041-9 PMC4455052 26045719

[36] Thibaud-Nissen, F., Souvorov, A., Murphy, T. D., DiCuccio, M. & Kitts, P. P8008 the NCBI eukaryotic genome annotation pipeline. *Journal of Animal Science* **94**, 184–184, 10.2527/jas2016.94supplement4184x (2016).

[37] Simão, F. A., Waterhouse, R. M., Ioannidis, P., Kriventseva, E. V. & Zdobnov, E. M. BUSCO: assessing genome assembly and annotation completeness with single-copy orthologs. *Bioinformatics* **31**, 3210–3212, 10.1093/bioinformatics/btv351 (2015). 26059717 10.1093/bioinformatics/btv351

[38] Manni, M., Berkeley, M. R., Seppey, M., Simão, F. A. & Zdobnov, E. M. BUSCO update: novel and streamlined workflows along with broader and deeper phylogenetic coverage for scoring of eukaryotic, prokaryotic, and viral genomes. *Mol. Biol. Evol.* **38**, 4647–4654, 10.1093/molbev/msab199 (2021). 34320186 10.1093/molbev/msab199 PMC8476166

[39] Kriventseva, E. V. *et al*. OrthoDB v10: sampling the diversity of animal, plant, fungal, protist, bacterial and viral genomes for evolutionary and functional annotations of orthologs. *Nucleic Acids Res.* **47**, D807–D811, 10.1093/nar/gky1053 (2019). 30395283 10.1093/nar/gky1053 PMC6323947

[40] Nevers, Y. *et al*. Quality assessment of gene repertoire annotations with OMArk. *Nat. Biotechnol.* **43**, 124–133, 10.1038/s41587-024-02147-w (2025). 38383603 10.1038/s41587-024-02147-w PMC11738984

[41] Uliano-Silva, M. *et al*. MitoHiFi: a python pipeline for mitochondrial genome assembly from PacBio high fidelity reads. *BMC Bioinformatics* **24**, 288, 10.1186/s12859-023-05385-y (2023). 37464285 10.1186/s12859-023-05385-y PMC10354987

[42] Allio, R. *et al*. MitoFinder: Efficient automated large-scale extraction of mitogenomic data in target enrichment phylogenomics. *Mol. Ecol. Resour.* **20**, 892–905, 10.1111/1755-0998.13160 (2020). 32243090 10.1111/1755-0998.13160 PMC7497042

[43] *Hyla annectans* mitochondrion, complete genome. *NCBI GenBank* http://identifiers.org/insdc:KM271781.1 (2019)

[44] Vertebrate Genomes Project. *Hyla sarda* genome assembly aHylSar1.hap2. *NCBI GenBank* http://identifiers.org/assembly:GCA_029493135.1 (2023).

[45] *Hyla sarda* isolate aHylSar1 mitochondrion, complete sequence, whole genome shotgun sequence. *NCBI GenBank* http://identifiers.org/insdc:CM056048.1 (2023)

[46] Formenti, G. *et al*. Gfastats: conversion, evaluation and manipulation of genome sequences using assembly graphs. *Bioinformatics* **38**, 4214–4216, 10.1093/bioinformatics/btac460 (2022). 35799367 10.1093/bioinformatics/btac460 PMC9438950

[47] Brown, M. R., Gonzalez de La Rosa, P. & Blaxter, M. tidk: a toolkit to rapidly identify telomeric repeats from genomic datasets. *Bioinformatics* **41**, btaf049, 10.1093/bioinformatics/btaf049 (2025). 39891350 10.1093/bioinformatics/btaf049 PMC11814493

