# Supplementary material for: Chromosome-level genome assembly of the Tyrrhenian tree frog (Hyla sarda)
Source: Sci Data. 2025 Sep 2;12:1539. doi: 10.1038/s41597-025-05760-9 (PMC12405506; doi:10.1038/s41597-025-05760-9)
Supplement: Supplementary file 1 — Supplementary Table 1 [file 41597_2025_5760_MOESM1_ESM.pdf]

Table S1. Genome statistics for 88 anuran genomes, retrieved from Genomes on a Tree (GoaT) using the search term, "tax_tree(8342[anurans])" on 15th February 2025. Genome size, chromosome number, and haploid number cells are coloured based on directly measured values (green) or values inferred from ancestral taxa (red). Assembly level is coloured by chromosome-level (green), scaffold-level (orange), or contig-level (red).

Original order	Scientific name	NCBI taxonomy ID	Genome size (bp)	Genome size (Gbp)	Chromosome number	Haploid number	Assembly span (bp)	Assembly span (Gbp)	Ratio of size:span	Assembly level
1	*Hyperolius nasutus*	143418	3726180000	3,73	24	12	2845579347	2,85	1,31	Scaffold
2	*Hyperolius riggenbachi*	752182	4630830000	4,63	24	12	4915935452	4,92	0,94	Chromosome
3	*Gastrophryne carolinensis*	8429	4762860000	4,76	22	11	4336248441	4,34	1,10	Chromosome
4	*Phrynobatrachus natalensis*	327965	4058700000	4,06	18	9	2031217451	2,03	2,00	Scaffold
5	*Phrynobatrachus minutus*	467733	4058700000	4,06	16	8	1483874292	1,48	2,74	Scaffold
6	*Zhangixalus dugritei*	462321	4058700000	4,06	26	13	3360000000	3,36	1,21	Scaffold
7	*Rhacophorus kio*	493789	4058700000	4,06	26	13	2660000000	2,66	1,53	Scaffold
8	*Aquarana catesbeiana*	8400	7335000000	7,34	26	13	6377618952	6,38	1,15	Chromosome
9	*Staurois parvus*	386267	6283650000	6,28	26	13	3982222951	3,98	1,58	Scaffold
10	*Rana temporaria*	8407	4391220000	4,39	26	13	4111422596	4,11	1,07	Chromosome
11	*Rana muscosa*	160500	5222520000	5,22	26	13	10160103425	10,16	0,51	Chromosome
12	*Rana kukunoris*	670907	5222520000	5,22	24	12	4830730561	4,83	1,08	Chromosome
13	*Glandirana rugosa*	8410	7970700000	7,97	26	13	7628398632	7,63	1,04	Scaffold
14	*Lithobates sylvaticus*	45438	5701740000	5,70	26	13	5150352190	5,15	1,11	Chromosome
15	*Lithobates pipiens*	8404	6337440000	6,34	26	13	5897899108	5,90	1,07	Contig
16	*Lithobates septentrionalis*	190274	7980480000	7,98	26	13	7098362355	7,10	1,12	Contig
17	*Lithobates clamitans*	145282	7012260000	7,01	26	13	6355615356	6,36	1,10	Contig
18	*Ptychadena erlangeri*	1342833	2552580000	2,55	24	12	1526448469	1,53	1,67	Scaffold
19	*Ptychadena robeensis*	2829185	2552580000	2,55	24	12	1591013924	1,59	1,60	Chromosome
20	*Conraua beccarii*	2790342	4058700000	4,06	26	13	1759549356	1,76	2,31	Scaffold
21	*Nanorana parkeri*	125878	4058700000	4,06	26	13	2053849526	2,05	1,98	Scaffold
22	*Hoplobatrachus occipitalis*	127645	5584380000	5,58	52	13	1909036378	1,91	2,93	Scaffold
23	*Phrynoglossus myanhessei*	2798809	4058700000	4,06	26	13	1829122027	1,83	2,22	Contig
24	*Pyxicephalus adspersus*	30357	1369200000	1,37	26	13	1563367516	1,56	0,88	Chromosome
25	*Platyplectrum ornatum*	2741728	2273850000	2,27	22	11	1065311793	1,07	2,13	Scaffold
26	*Mixophyes australis*	3058680	2273850000	2,27	24	12	3127878685	3,13	0,73	Scaffold
27	*Mixophyes fleayi*	3061075	2273850000	2,27	24	12	2969628263	2,97	0,77	Chromosome
28	*Limnodynastes dumerilii*	104065	3178500000	3,18	22	11	2378679715	2,38	1,34	Scaffold
29	*Taudactylus pleione*	3041213	2024459999	2,02	24	12	5507797579	5,51	0,37	Contig
30	*Pseudophryne corroboree*	495146	8557500000	8,56	24	12	8872758793	8,87	0,96	Chromosome
31	*Boana sibleszi*	2668082	3418110000	3,42	24	12	1956429660	1,96	1,75	Scaffold
32	*Pseudis tocantins*	428384	1506120000	1,51	24	12	24258155	0,02	62,09	Scaffold
33	*Scinax staufferi*	318396	2934000000	2,93	24	12	1455304001	1,46	2,02	Scaffold
34	*Dendropsophus ebraccatus*	150705	2317860000	2,32	30	15	2214937069	2,21	1,05	Chromosome
35	*Pseudacris regilla*	47562	3726180000	3,73	24	12	3600278088	3,60	1,03	Scaffold
36	*Hyla sarda*	327740	4312979999	4,31	26	13	4142159359	4,14	1,04	Chromosome
37	*Smilisca baudinii*	279982	4260412499	4,26	24	12	1457742877	1,46	2,92	Scaffold
38	*Triprion petasatus*	317403	4260412499	4,26	24	12	1769779451	1,77	2,41	Scaffold
39	*Tlalocohyla loquax*	317335	4260412499	4,26	24	12	1743827571	1,74	2,44	Scaffold
40	*Osteocephalus taurinus*	248862	2347200000	2,35	24	12	1846173392	1,85	1,27	Scaffold
41	*Trachycephalus venulosus*	213803	3276300000	3,28	24	12	1491269799	1,49	2,20	Scaffold
42	*Phyllomedusa bahiana*	860369	6518370000	6,52	52	26	4741016426	4,74	1,37	Contig
43	*Eleutherodactylus coqui*	57060	3794640000	3,79	26	13	3371297528	3,37	1,13	Chromosome
44	*Oophaga pumilio*	51950	8753100000	8,75	20	10	3493380730	3,49	2,51	Scaffold
45	*Oophaga sylvatica*	152496	8753100000	8,75	20	10	5186611867	5,19	1,69	Scaffold
46	*Ranitomeya imitator*	111125	8753100000	8,75	18	10	5956610564	5,96	1,47	Chromosome
47	*Phyllobates terribilis*	111132	8753100000	8,75	24	12	4220363320	4,22	2,07	Scaffold
48	*Dendrobates tinctorius*	92724	8753100000	8,75	18	9	6355763190	6,36	1,38	Contig
49	*Allobates femoralis*	92733	6953580000	6,95	24	12	5324439307	5,32	1,31	Scaffold
50	*Rhinella marina*	8386	4655280000	4,66	22	11	3473313001	3,47	1,34	Scaffold
51	*Bufotes viridis*	30338	4723740000	4,72	22	11	3796774568	3,80	1,24	Chromosome
52	*Incilius valliceps*	8388	4498800000	4,50	22	11	1945516919	1,95	2,31	Scaffold
53	*Anaxyrus boreas*	30325	5183400000	5,18	22	11	5278819005	5,28	0,98	Scaffold
54	*Sclerophrys regularis*	1978144	3902220000	3,90	20	10	1762966613	1,76	2,21	Scaffold
55	*Sclerophrys asmarae*	1978132	4880220000	4,88	40	20	1767022399	1,77	2,76	Scaffold
56	*Atelopus laetissimus*	2776728	5095380000	5,10	22	11	3506202348	3,51	1,45	Contig
57	*Bufo gargarizans*	30331	5476800000	5,48	22	11	4545465442	4,55	1,20	Chromosome
58	*Bufo bufo*	8384	6454800000	6,45	22	11	5044744194	5,04	1,28	Chromosome
59	*Adenomera lutzi*	1133662	3141825000	3,14	26	13	1871846158	1,87	1,68	Scaffold
60	*Leptodactylus melanonotus*	228420	2464560000	2,46	22	11	1447694148	1,45	1,70	Scaffold
61	*Leptodactylus mystaceus*	323527	3141825000	3,14	22	11	1397553186	1,40	2,25	Scaffold
62	*Leptodactylus fragilis*	349990	3141825000	3,14	22	11	1474876193	1,47	2,13	Scaffold
63	*Leptodactylus fuscus*	238119	2591700000	2,59	22	11	2307711212	2,31	1,12	Chromosome
64	*Leptodactylus fallax*	375434	3141825000	3,14	22	11	2510271426	2,51	1,25	Scaffold
65	*Engystomops pustulosus*	76066	2366760000	2,37	22	11	2158203323	2,16	1,10	Chromosome
66	*Discoglossus pictus*	8351	5134500000	5,13	28	14	3872581802	3,87	1,33	Chromosome
67	*Xenopus tropicalis*	8364	1701720000	1,70	20	10	1451283599	1,45	1,17	Chromosome
68	*Xenopus laevis*	8355	3080700000	3,08	36	18	2730434022	2,73	1,13	Chromosome
69	*Xenopus borealis*	8354	3481680000	3,48	36	18	2747450723	2,75	1,27	Chromosome
70	*Xenopus petersii*	288555	3542805000	3,54	36	18	2883778474	2,88	1,23	Chromosome
71	*Pipa parva*	247091	2376540000	2,38	30	15	1370893480	1,37	1,73	Scaffold
72	*Pipa carvalhoi*	191480	2376540000	2,38	20	10	1192124124	1,19	1,99	Contig
73	*Hymenochirus boettgeri*	247094	2376540000	2,38	24	12	3210867089	3,21	0,74	Chromosome
74	*Rhinophrynus dorsalis*	43566	4684620000	4,68	22	11	4745552136	4,75	0,99	Chromosome
75	*Leptobrachium leishanense*	445787	2841090000	2,84	26	13	3549060464	3,55	0,80	Chromosome
76	*Leptobrachium ailaonicum*	428466	2841090000	2,84	26	13	3535795546	3,54	0,80	Chromosome
77	*Pelodytes ibericus*	233781	2303190000	2,30	24	12	2077641644	2,08	1,11	Chromosome
78	*Scaphiopus couchii*	85089	1046460000	1,05	26	13	484148731	0,48	2,16	Scaffold
79	*Scaphiopus holbrookii*	8435	1369200000	1,37	26	13	710849620	0,71	1,93	Scaffold
80	*Pelobates fuscus*	191477	4371660000	4,37	26	13	3606456729	3,61	1,21	Chromosome
81	*Pelobates cultripes*	61616	4122270000	4,12	26	13	3094940563	3,09	1,33	Chromosome
82	*Spea multiplicata*	30317	1310520000	1,31	26	13	1075972709	1,08	1,22	Scaffold
83	*Spea bombifrons*	233779	1207830000	1,21	26	13	987655159	0,99	1,22	Chromosome
84	*Spea hammondii*	228670	1413210000	1,41	26	13	1155539324	1,16	1,22	Scaffold
85	*Ascaphus truei*	8439	4068479999	4,07	46	23	3722616900	3,72	1,09	Chromosome
86	*Bombina variegata variegata*	191472	10132080000	10,13	24	12	4679106955	4,68	2,17	Scaffold
87	*Bombina variegata*	8348	8958480000	8,96	24	12	9369772323	9,37	0,96	Chromosome
88	*Bombina bombina*	8345	11134530000	11,13	24	12	10019663791	10,02	1,11	Chromosome
